# Silent Threat: Bilateral Giant Asymptomatic Endometriotic Cysts With Unilateral Sudden Rupture—A Case Report

**DOI:** 10.1155/crog/1739044

**Published:** 2025-06-25

**Authors:** Muhammad Dwi Priangga, Adhitya Yudha Maulana, Yasmine Syifa Nabila Budi, Syauqi Maulana Idhar, Herbert Situmorang

**Affiliations:** ^1^Department of Obstetrics and Gynecology, Faculty of Medicine, Cipto Mangunkusumo Hospital, University of Indonesia, Jakarta, Indonesia; ^2^Obstetric and Gynecology Resident, Faculty of Medicine, University of Indonesia, Jakarta, Indonesia; ^3^Department of Obstetrics and Gynecology, Faculty of Medicine, University of Indonesia, Jakarta, Indonesia

## Abstract

Endometriotic cysts are common, but bilateral giant endometriosis cyst with asymptomatic cases are extremely rare. Rupture is also uncommon, yet when it occurs, it can mimic appendicitis or ectopic pregnancy due to peritoneal irritation, often requiring emergency intervention. A 25-year-old woman presented with nausea, vomiting, and an enlarging abdominal lump. Ultrasonography revealed bilateral ovarian cystic masses with ground glass appearances and free subhepatic fluid. Due to worsening symptoms despite initial resuscitation, emergency exploratory laparotomy was performed. Intraoperatively, a ruptured right ovarian cyst (20 × 15 × 15 cm) with extensive adhesions to the posterior uterus, rectum, and right ovarian fossa was identified, along with a left ovarian endometriotic cyst (12 × 10 × 10 cm). The patient underwent right salpingo-oophorectomy, left cystectomy, and ureterolysis. Histopathology confirmed endometriotic cysts. Rapid surgical intervention is crucial in ruptured cysts to minimize adhesion formation and preserve fertility. Postoperatively, continuous hormonal therapy, such as oral progesterone or an intrauterine device, is recommended to decrease recurrence.

## 1. Introduction

Endometriotic cysts are a subtype of pathological ovarian cysts resulting from old hemorrhages due to endometriosis [1, 2]. These cysts, known as “chocolate cysts,” contain thick, brown fluid resembling a “chocolate” substance [1, 2]. However, most endometriotic cysts typically range from 1 to 6 cm in size, while giant ovarian endometriomas are exceptionally rare, with a reported frequency of less than 1% and only a few documented cases [3, 4]. There is no definitive cutoff for giant endometriomas, but some studies classify endometriotic cysts exceeding 10–15 cm as giant endometriomas [5]. Although ovarian endometriosis is prevalent among women of reproductive age, ruptured endometriotic cysts are uncommon. However, when they occur, they can cause severe abdominal pain and chemical peritonitis, often necessitating emergency surgery [6, 7]. Symptoms of a ruptured endometriotic cyst include severe abdominal pain, nausea, vomiting, muscle cramps, and rebound tenderness, often resembling other conditions like appendicitis or ruptured ectopic pregnancy [1]. Diagnosis typically involves a transvaginal ultrasound to detect free fluid similar to that of an endometriotic cyst. Emergency surgery for ruptured endometriotic cysts is challenging due to potential adhesions and fertility preservation concerns, but long-term progesterone therapy can help to decrease the postoperative recurrence of endometriosis [2, 7].

## 2. Case Report

A 25-year-old patient presented to the emergency department with the chief complaint of nausea and vomiting for the last 8 h prior to admission. The patient also reported a lump in the abdominal area for the past year, which had progressively increased in size over the last month. There was no history of bleeding from the birth canal, dysmenorrhea, dyspareunia, bowel or bladder disorders, or weight loss. The patient denied any history of other illnesses. She had menarche at the age of 12, with regular menstruation lasting 5–7 days per cycle, using two to three pads per day, and without dysmenorrhea. The patient has been married for a year, and no contraceptive device usage was reported.

**Figure 1:**
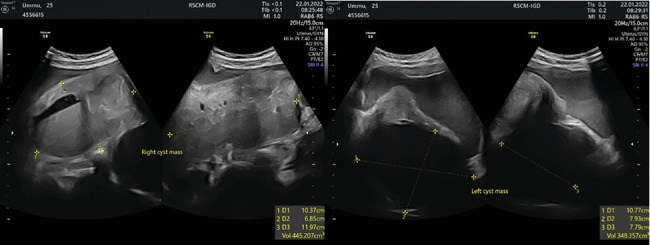
Ultrasonographic examination on right and left ovaries showing cystic mass.

Physical examination result shows palpable right and left adnexal masses and weak parametrium. Ultrasonographic examination on the right ovary shows a cystic mass with ground glass appearance, with a size of 103 × 68 × 119 mm, no papillary appearance, solid part, and vascularity. Ultrasonographic examination on the left ovary also shows a cystic mass with ground glass appearance, with a size of 107 × 79 × 77 mm, no vascularity (Figure 1). Free subhepatic fluid was also noted. Laboratory examination shows thrombocytosis (503,000/*μ*L), leucopenia (1900/*μ*L), elevated liver enzymes (ALT/AST 90/152), and hyponatremia (130 mEq/L). Other laboratory examinations were within normal limits.

Patient was assessed with bilateral cystic ovarian neoplasm with solid part, hypovolemic shock Grade II due to vomitus, thrombocytosis, leucopenia, elevated liver enzymes, hyponatremia, and ascites. Initial treatment includes fluid resuscitation (1000 mL Ringer lactate loading followed by 500 mL/8 h maintenance), 3 × 8 mg ondansetron IV, 3 × 40 mg omeprazole IV, and 3 × 500 mg NaCl caps orally.

Patient's condition was not improved after initial treatment. Sinus tachycardia was also present, with inadequate improvement after fluid resuscitation. The patient was then consulted to Tropical Infection and Cardiology Division for further examination. The patient condition worsens during assessment, with blood pressure of 89/63 mmHg, HR 148×/min, and RR 24×/min. Physical examination reveals a distended abdomen with positive abdominal defans muscular, no active bleeding from the vulva and vagina. After performing puncture of the abdomen, it produces a thick, brown chocolate fluid corresponding to endometriotic cyst content. The patient was then assessed with acute abdomen due to suspected hemoperitoneum due to bilateral cyst rupture. The patient was then planned for emergency exploratory laparotomy.

**Figure 2:**
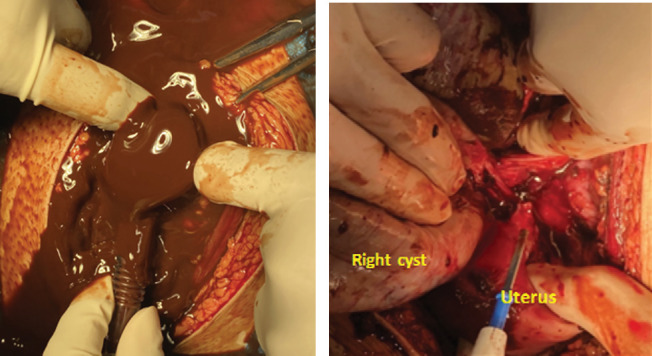
(a) Thick, brown fluid was noted upon incision of the peritoneum. (b) Cyst wall perforation.

**Figure 3:**
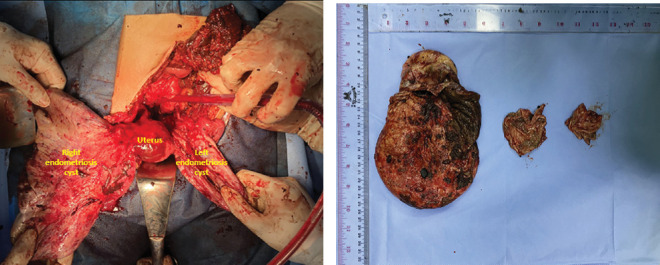
(a) Left and right cysts during exploratory laparotomy. (b) Left and right cysts after evacuation.

A laparotomy was performed and thick, brown fluid was noted upon incision of the peritoneum. Approximately 3000 mL of liquid was evacuated using suction (Figure 2a). Upon exploration, the fluid came from a ruptured right ovarian cyst with a size of 20 × 15 × 15 cm, smooth surface, with adhesion to the posterior uterus, rectum, and right ovarian fossa (Figure 2b). Adhesiolysis was performed, and a diagnosis of ruptured right endometriosis cyst was made. There was also a cyst on the left ovary with a size of 12 × 10 × 10 cm with adhesion to the left ovarian fossa. Adhesiolysis was performed, and thick, brown fluid was noted upon the procedure. Therefore, the patient was diagnosed with bilateral giant endometriosis cyst. Right salpingo-oophorectomy and left cystectomy were performed (Figure 3a,b), and the specimen was sent for histopathology examination. Further exploration towards the right ureter was performed by opening the right anterior broad ligament at the level of the right infundibulopelvic ligament. Adhesion of the mass to the right ureter was noted and ureterolysis was performed. After ensuring there was no active bleeding, the abdomen was closed.

The patient was then admitted to the intensive care unit (ICU) for observation. The patient's vital status was stable with hypoalbuminemia. Intravenous albumin 25% was administered, and the patient was given high-protein diet. After 1 day in the ICU, the patient was then transferred to the obstetric and gynecologic ward. Three days after surgery, the patient was discharged. Postoperative Day 14, the patient came to the gynecology clinic bringing the histopathology result. The result confirms the diagnosis of an ovarian endometriotic cyst. The patient was then planned for progesterone therapy to decrease endometriosis recurrence.

## 3. Discussion

Ovarian endometriotic cysts (chocolate cyst) are the most common type of endometriosis, accounting for approximately 80% of all endometriosis. The pathophysiology behind this type of cyst is still unknown, although there are several hypotheses that have been formulated [6]. The most popular hypothesis is the retrograde menstruation hypothesis. In this hypothesis, menstrual blood, along with endometrial cells, travels back through the fallopian tube into the ovarium, seeding the ovarium with endometrial cells. During the menstrual cycle, these cells gradually bleed, and when drainage is not adequate, they slowly develop into a cyst [1, 6]. The cyst grows during menstruation, and when it is large enough, the tension within the cyst is too high, leading to rupture [8].

There is no universally established cutoff diameter for defining a “giant” endometriotic cyst. However, various studies suggest that endometriomas exceeding 10–15 cm are considered rare and noteworthy, highlighting the exceptional nature of larger cysts. These larger cysts often attract attention due to their infrequency and potential complications. The symptoms range from menstrual issues like pain and heavy bleeding to more general abdominal problems including pain, bloating, swelling, and potentially serious conditions indicated by malaise, nausea, anorexia, chills, and constipation [3–5, 9, 10].

Rupture of an ovarian endometriotic cyst is a rare occurrence, only occurring in less than 3% of women with this condition. The rupture occurs more often when the cyst is ≥ 6.0 cm in diameter, and during pregnancy, due to hormonal stimulation of endometrial stromal elements [11]. In our case, however, rupture occurs outside of pregnancy. This is most probably caused by the large cyst size (20 cm), which substantially increases the risk of cyst rupture.

The gold standard for diagnosis in these cases is exploratory laparotomy, although several imaging techniques may be performed to confirm the suspicion of a ruptured ovarian endometriotic cyst. Transvaginal ultrasonography (TVUS) is the most common imaging modality in this case. TVUS of a ruptured ovarian endometrial cyst shows an ovarian cyst with heterogeneous content, irregular contour, parietal discontinuity, and free abdominal fluid. However, in smaller cysts, these findings may be wrongly interpreted as other gynecological conditions such as ectopic pregnancy or spontaneous hemoperitoneum [11]. In our patient, the cyst was easily seen due to its large size. However, the free fluid in the peritoneal space was interpreted as ascites due to the presence of elevated liver enzymes as well. Therefore, the differential diagnosis of a ruptured endometriotic cyst should be considered when there is free abdominal fluid in the presence of an endometriotic cyst.

Once diagnosis is made, prompt treatment must be administered for endometriotic cyst as untreated cyst may cause pelvic adhesion, induce new endometriosis, and subsequently reduce fertility or even trigger infertility. In patients of childbearing age, surgical removal of the cyst is the preferred treatment as it conserves endocrine function and fertility. Surgical removal should include cystectomy, electrocautery for endometriotic lesion, myomectomy when necessary, and adhesiolysis when necessary [6]. In smaller cysts, however, watchful waiting could be done. The general consensus is that cysts up to 3 cm in diameter should be left untreated [12].

Although surgery is the preferred treatment modality, the recurrence rate for these cysts is considerably high. Removal of the content of the cyst using ultrasound-guided puncture has the highest recurrence rate, with up to 90% of patients reporting recurrence of the cyst 6 months after the procedure. Surgical removal of the cyst, involving adhesiolysis, cystectomy, irrigation of the cyst, and excision of the cyst wall, is proven to have a lower recurrence rate of approximately 10%–40%. The recurrence rate is also associated with the severity of endometriosis, although the prognostic factor is poor and not all studies support this theory. Other factors that affect recurrence include bilateral cyst, larger cyst size, and younger age of onset [12].

Laparotomy and laparoscopy each have distinct advantages in the management of giant endometriosis. Laparotomy is preferred for complex cases with extensive adhesions or bowel involvement as it provides excellent surgical access and allows thorough resection, though it carries higher risks of postoperative complications, a longer recovery period, and greater costs [13, 14]. In contrast, laparoscopy, being minimally invasive, offers faster recovery, less blood loss, a lower risk of complications, and better fertility outcomes, although its access is limited for severe cases [15]. The choice of surgical method should be tailored to the patient's condition, the severity of endometriosis, and the surgeon's expertise. In our patient's case, laparotomy was more suitable due to the large size of the cyst (20 cm), the suspicion of rupture after abdominal puncture, which revealed thick, brown chocolate fluid, and signs of peritonitis in the patient. These factors necessitated a more open and accessible approach for safe and complete cyst removal. Additionally, the patient's condition and the complexity of the surgery made laparotomy the safer and more reliable choice. Current evidence shows that pharmacotherapy using oral contraceptives is efficient in controlling menstrual pain and in decreasing the recurrence of endometriotic cyst. Research by Seracchioli et al. [16] shows that the usage of both combination and continuous contraceptives significantly lowers the recurrence of endometriotic cyst (29% vs. 14.7%). A systematic review by Zorbas et al. [17] also shows that while both cyclic and continuous contraceptives significantly lower recurrence, continuous oral contraceptives provide a better effect and lower recurrence rate of endometriotic cyst. In addition, continuous oral contraceptive containing progestin also decreases the amount of menstruation, thereby decreasing retrograde menstruation and reducing the chance of endometriosis recurrence since it directly inhibits the pathophysiology of endometriosis [18].

In terms of progestin delivery, several methods have already been researched, including oral, subcutaneous, intramuscular, intrauterine, and vaginal routes. Two progestins, norethisterone acetate 2.5–5.0 mg/day and dienogest 2 mg/day, are the most used progestins for the treatment of endometriosis [18]. Another possible alternative is using a hormonal intrauterine device with embedded levonorgestrel. These devices abolish menstruation in approximately one-third of users or significantly decrease the amount of bleeding in two-thirds of users. Additionally, they do not require daily maintenance, which further increases treatment compliance. Moreover, these devices can be inserted at the end of surgery, making their use easier [18].

As the most common complication for endometriosis is infertility, treatment for restoring fertility must be considered. One of the most common treatments is surgical restoration of altered anatomy of the uterus. These procedures consisted of adhesiolysis of the lesion and removal of the endometriotic lesion. If after restoration, fertility is not restored, IVF can be performed to bypass adhesion and tubal patency problems [18].

## 4. Conclusion

This case highlights the extremely rare occurrence of a bilateral asymptomatic giant endometriotic cyst with unilateral sudden rupture in a 25-year-old woman presenting with nonspecific symptoms of peritonitis, which may be misdiagnosed as appendicitis or ectopic pregnancy. This case underscores the importance of considering ruptured endometriotic cysts in the differential diagnosis of acute abdomen, especially in the presence of a palpable abdominal mass and free fluid. Laparotomy remains the gold standard for diagnosis and treatment, with cystectomy and adhesiolysis being crucial for fertility preservation in younger patients. Postoperative management with progesterone therapy is essential to minimize recurrence, and future fertility-sparing treatments like IVF may be necessary depending on the patient's reproductive goals.

## Data Availability

Data and materials are available when needed by contacting the corresponding author.
